# Self-esteem is associated with health status and PROMs in advanced age independent of multidimensional frailty: secondary analysis from a RCT with 6-month follow-up

**DOI:** 10.1007/s10433-025-00888-4

**Published:** 2025-10-30

**Authors:** Anna Maria Affeldt, Luisa Mück, Ingrid Becker, Anne Ferring, Jill Stegemann, Laura Wiebe, Thomas Benzing, Malte P. Bartram, M. Cristina Polidori

**Affiliations:** 1 Department II of Internal Medicine and Centre for Molecular Medicine Cologne, Faculty of Medicine and University Hospital Cologne, University of Cologne, Kerpener Straße 62, 50937 Cologne, Germany; 2 Institute of Medical Statistics and Computational Biology, Faculty of Medicine, University of Cologne, Cologne, Germany; 3 CECAD, University of Cologne, Faculty of Medicine and University Hospital Cologne, Cologne, Germany

**Keywords:** Comprehensive geriatric assessment (CGA), Multidimensional Prognostic Index (MPI), Rosenberg Self-Esteem Scale (RSES), Patient-reported outcome measures (PROMs), Successful ageing

## Abstract

The predictive value of patient-reported outcome measures (PROMs), such as self-esteem in advanced age in relation to biological age (frailty), remains to be elucidated. In a secondary analysis of a randomised controlled trial (RCT), 107 hospitalised patients (mean age 77.2 (SD 7.1) years, 56% female) underwent a comprehensive geriatric assessment (CGA). Assessments included the Multidimensional Prognostic Index (MPI), the Rosenberg Self-Esteem Scale (RSES), evaluation of geriatric syndromes and resources, the EQ-5D-5L quality-of-life scale, and the Geriatric Depression Scale (GDS). Follow-up data were collected via telephone six months later. After adjusting for age, sex, intervention, and MPI, baseline RSES were significantly associated with pressure ulcer risk (*p* = 0.009), nutritional status (*p* = 0.042), number of geriatric syndromes (*p* = 0.003), geriatric resources (*p* < 0.001), depressive symptoms (GDS, *p* < 0.001), and quality of life (EQ-5D-5L, *p* = 0.020). These findings show that self-esteem appears to be an independent mediator of multiple geriatric outcomes, including geriatric resources and syndromes, as well as of PROMs, beyond what is explained by MPI-based frailty. Incorporating self-esteem measures into CGA may enhance the identification of at-risk individuals and guide interventions.

**Supplementary Information:**

The online version contains supplementary material available at 10.1007/s10433-025-00888-4.

## Introduction

Self-esteem plays a significant role throughout the lifespan, influencing an individual’s capacity to lead a successful and fulfilling life, as well as their psychological well-being (Rosenberg 1965). As defined by Rosenberg, self-esteem can be understood as “one’s positive or negative attitude toward the self and an overall evaluation of one’s worth and value” (Rosenberg 1965). In contrast, self-efficacy can be conceptualised as “the belief that one has the power to effect changes by one’s own actions” (Bandura 2004). Latent growth curve analyses indicate that self-esteem follows a quadratic trajectory across the adult lifespan, reaching a peak at approximately 60 years of age and subsequently declining in old age (Orth et al. 2010). The findings of the longitudinal study of generations indicate that self-esteem should be conceptualised as a causal factor rather than an outcome of life experiences. This suggests that self-esteem exerts an influence on the lifespan trajectories of affect, depression, and health (Orth et al. 2012), as well as quality of life (Tavares et al. 2016).

Given that frailty, defined as a multidimensional geriatric syndrome characterised by the loss of individual reserves and increased vulnerability to internal and external stressors (Hoogendijk et al. 2019), often plays a significant role in the outcomes of older individuals, it remains unclear whether self-esteem is a crucial mediator and predictor that interacts with these secondary outcomes independently of frailty.

The available evidence suggests that older adults with high levels of self-esteem demonstrate a reduced likelihood of experiencing loss and minimal fluctuations in their self-esteem, which may serve as further evidence of resilience in older adults (Collins and Smyer 2005). This is exemplified by the observation that diminished self-esteem is correlated with heightened stress perception and stress reactivity (Galanakis et al. 2016; Amestoy et al. 2023). Conversely, higher self-esteem is associated with lower reactivity to stressful events. Individuals with higher self-esteem have been demonstrated to exhibit greater resilience and lower distress levels compared to those with lower self-esteem (Brown 2010). It is well documented that chronic stress, or frequent intermittent stress, plays a pivotal role in the development and maintenance of a multitude of physical and psychological health ailments across the lifespan, with increased vulnerability in later life (Gouin et al. 2008; Blazer and Hybels 2005; Mohammadi et al. 2022). Furthermore, individuals who are chronically stressed tend to age rapidly, a phenomenon known as accelerated ageing (Yegorov et al. 2020). This is attributed to a systemic increase in oxidative stress and a reduction in antioxidant capacity, which ultimately leads to multisystem dysfunction and frailty (Assar et al. 2020).

It is of concern that key factors influencing resilience-based functional independence, such as psychosocial elements, are frequently overlooked in the context of high-performance medicine for older patients, who are prone to readmission and disability (Zamir et al. 2006). A well-established method for diagnosing and treating older patients in a holistic manner is the utilisation of a comprehensive geriatric assessment (CGA) (Ellis et al. 2017; Rubenstein et al. 1984). This approach enables the identification of geriatric resources and geriatric syndromes, among other benefits (Meyer et al. 2020).

The CGA-based measurement of the Multidimensional Prognostic Index (MPI) is currently the most reliable and highly validated tool for calculating mortality risk. The MPI may be considered a proxy for frailty and biological age (Zampino et al. 2022). It has been demonstrated to be significantly associated with patient-reported outcome measures (PROMs) concerning health-related quality of life (HRQoL), as assessed by the European Quality of Life-5 Dimensions (EQ-5D-5L) (Rarek et al. 2021; Heeß et al. 2022), and enables the quantification of patients’ multidimensional frailty and prognosis (Pilotto et al. 2008; Bureau et al. 2017; Dent et al. 2016; Meyer et al. 2019; Warnier et al. 2016). Recently, it has been demonstrated that there is a significant association between the MPI and self-esteem as measured by the Rosenberg Self-Esteem Scale (RSES) (Meyer et al. 2022).

In the light of the recent observation that a tailored intersectoral discharge programme (TIDP) is able to improve self-confidence as part of self-esteem, together with frailty and prognosis in older inpatients undergoing high-performance medicine and usual rehabilitative treatment (Meyer et al. 2022), and in view of the substantial lack of information on the prognostic role of self-esteem in patients with or at risk of frailty, the primary objective of the present secondary analysis was to investigate the predictive value of self-esteem in terms of mortality, independent of chronological age, MPI-biological age, and biological sex in older patients of a geriatric ward (Meyer et al. 2022). Secondary outcomes included the investigation of the relationship between self-esteem and health status, the incidence of rehospitalisation within a six-month period, and the utilisation of patient-reported outcome measures (PROMs).

## Material & methods

### Transparency and openness

The study, entitled "Vun nix kütt nix", on which this secondary analysis is based, was approved by the Ethical Committee (EK number 18-394) of the University Hospital Cologne, Germany, and a signed informed consent form was obtained from each patient or proxy. The study is registered at the German Clinical Trials Register (DRKS-ID: DRKS00015996) and adheres to the ethical principles for medical research involving human subjects as outlined in the Declaration of Helsinki (1983).

The raw and/or processed data on which the secondary analysis conclusions are based, as well as the syntax required to reproduce the analyses, are available upon request from the authors.

### Patients

This secondary analysis is based on data from a randomised controlled trial conducted between October 2019 and August 2020 in 110 patients aged 60 years and older who were hospitalised for an acute illness and undergoing simultaneous usual rehabilitative care by means of an early complex geriatric rehabilitation programme, as previously described (Meyer et al. 2022). The study population consisted of white individuals of both sexes. In addition to the age criterion of over 60 years, the inclusion criteria comprised multimorbidity (defined as the co-occurrence of two or more chronic medical conditions requiring treatment (Akker et al. 2009)), admission for the treatment of an acute disease, the presence of at least two geriatric syndromes requiring usual rehabilitative care, and the ability to provide informed consent. Patients were excluded if they had a life-threatening condition, severe disability, or neuropsychiatric disorders that impaired their communication abilities (Meyer et al. 2022). Patients were randomly assigned to either the intervention or control group in a 1:1 ratio. The TIDP group received an interprofessional discharge treatment plan, which consisted of multidimensional personalised counselling and a guidebook. Subsequent to the 1-, 3-, and 6-month mark, patients were contacted via telephone to furnish information, including data regarding survival, readmissions, mood as gauged by the Geriatric Depression Scale (GDS), quality of life as assessed by EQ-5D-5L, and self-esteem as evaluated by RSES (Meyer et al. 2022).

**Fig. 1:**
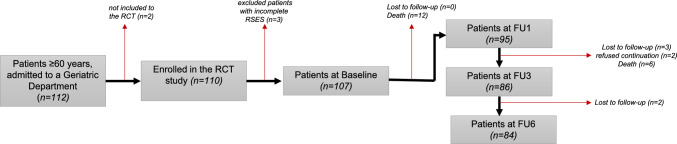
Study flow chart. *Notes*: RCT = randomised controlled trial; FU = follow-up; RSES = Rosenberg Self-Esteem Scale

In the context of the present investigation, patients with incomplete RSES data (n = 3) were excluded from the subsequent analysis. The final sample size comprised 107 patients (57 patients included in the TIDP, 50 patients in the control group), as shown in Fig. 1. The discrepancies in the number of follow-up data collected in Table 1**, Supp. Table 1–2,** are attributable to the patients’ willingness to respond.

**Table 1:** Patient characteristics of the cohort at baseline

	N (%) *(n* = *107)*
Age (years), *mean (SD)*	77.2 (7.1)
Female	60 (56)
Family status	
Married	59 (55.1)
Widowed	28 (26.2)
Single	20 (18.7)
Education (years), *median (IQR), n* = *105*	12.0 (5.0)
Ambulant care	27 (25.2)
Grade of care°*, n* = *106*	
None	47 (44)
1	2 (2)
2	29 (27)
3	21 (20)
4	7 (7)
Number of main diagnoses, *median (IQR),* n = 106	8 (5)
Hospitalised in the year before*,* n = 105	84 (80)
Number of medications*, mean (SD)*	10.6 (4.5)
Length of Stay, *median (IQR)*	17 (8)
RSES, *median (IQR)*	26 (5)

RSES = Rosenberg’s Self-Esteem Scale; °grade of care (measurement of nursing needs as measured by the German nursing care insurance grade 0 to 5, with 0 indicating no dependence in terms of need for care and 5 displaying highest need of care (Baake et al. 2017))

*Of the n* = *110 patients enrolled in the RCT study (17), a subsample of n* = *107 patients with complete RSES assessment at baseline was used for analyses. The flow chart illustrates the number of patients included in the follow-up analyses and provides information about the number and reason for exclusion.*

### Assessments

The RSES is comprised of ten items, five of which pertain to self-confidence and five to self-deprecation. The items are rated on a four-point Likert scale (3 = completely agree; 2 = rather agree; 1 = rather disagree; 0 = completely disagree for positive statements and vice versa for negative statements), resulting in a total score from zero to 30 points (Roth et al. 2008). A higher score on the scale is indicative of a higher level of self-esteem.

Additional data were gathered through the utilisation of the CGA and MPI calculation. The MPI is based on six assessments, including: the following instruments were employed: the Cumulative Illness Rating Scale (CIRS) (Linn et al. 1968), the Exton Smith Scale (ESS) (Bliss et al. 1966), the Mini Nutritional Assessment Short Form (MNA-SF) (Sancarlo et al. 2011), Katz’s Activities of Daily Living (ADL) (Katz et al. 1970), Lawton’s Instrumental Activities of Daily Living (IADL) (Lawton and Brody 1969), and the Short Portable Mental Status Questionnaire (SPMSQ) (Pfeiffer 1975), in addition to the number of drugs taken and living conditions (Pilotto et al. 2008). The MPI is a continuous variable that indicates mortality risk and the degree of multidimensional frailty. It ranges from 0, which represents the lowest risk and greatest robustness, to 1, which represents the highest risk and most severe frailty (Pilotto et al. 2008). The system allows for the classification of individuals into one of three mortality risk/frailty grades. MPI-1 is indicative of low risk and robustness, with a score ranging from 0 to 0.33. MPI-2 is associated with intermediate risk and prefrailty, with a score between 0.34 and 0.66. MPI-3 is indicative of severe risk and frailty, with a score ranging from 0.67 to 1 (Hoogendijk et al. 2019; Pilotto et al. 2008; Dent et al. 2019).

Additionally, the GDS (Yesavage et al. 1982) and the HRQoL (Guyatt et al. 1993) were evaluated using the EQ-5D-5L (Herdman et al. 2011) and a visual analogue scale (VAS). Additionally, a total of 17 geriatric syndromes were assessed (including incontinence, instability, immobility, cognitive impairment, inanition, impoverishment, polypharmacy, depression or irritability, sensorial impairment, insomnia, irritable colon, incoherence/delirium, iatrogenic disease, chronic pain, social isolation, swallowing disorder, and fluid/electrolyte imbalance). Furthermore, ten geriatric resources were identified (good living conditions and favourable situations in physical, social, economic, spiritual, motivational, emotional, mnestic, competence-related, and intellectual issues), as previously described (Meyer et al. 2020). Finally, main and secondary diagnoses, grade of care (measurement of nursing needs as measured by the German nursing care insurance grade 0 to 5, with 0 indicating no dependence in terms of need for care and 5 displaying the highest need of care (Baake et al. 2017)), the number of hospitalisations, and the incidence of falls in the previous year were documented.

### Statistical analysis

Descriptive statistics are expressed using absolute numbers and relative frequencies for the description of categorical variables and means (standard deviation, SD) or medians (interquartile range, IQR) for continuous and ordinal variables. 

The Kolmogorov–Smirnov test was employed to ascertain the suitability of the data for normal distribution.

As the aim was to examine whether there is a directional influence between variables, rather than simply the pure connection, linear and binary logistic regression analyses were employed to test the associations between self-esteem on admission and the clinical scales. These regression models were adjusted for chronological age, biological sex, TIDP intervention, and MPI-biological age on admission, unless otherwise specified. For assessment of multicollinearity, correlations were calculated, but none were significant. No variable selection was executed; results of the full models are shown. Clinical outcomes at the 1-month, 3-month, and 6-month marks were additionally adjusted for the corresponding scales at the time of admission whenever applicable. The graphical representations were generated using GraphPad Prism version 10.2.0 (392).

Two-tailed probabilities were reported, and a significance level of α = 0.05 was employed. All analyses are exploratory; therefore, p-values were not adjusted for multiple testing.

Since our intention was not prediction modelling, goodness-of-fit assessment or validation measures for regression models are not presented. The explanatory power (Nagelkerke’s R^2^) was throughout low, and we acknowledge the limitations inherent in small sample sizes. Future research with larger cohorts and external validation is warranted to confirm these findings.

All analyses were conducted using the Statistical Package for the Social Sciences (SPSS) software, version 28.0 (SPSS Inc., Chicago, IL).

## Results

### Demographic and clinical characteristics of the study population

The majority of the 107 patients were female (56%), with a mean age of 77.2 (SD 7.1) years. The median RSES score was 26 (IQR 5). The clinical characteristics are presented in Table 1. For n = 2 patients, information concerning the MPI was missing.

### Primary outcome: self-esteem and mortality

The overall mortality rate over the six-month follow-up period was 17% (*n* = 18). During the follow-up period, no significant association was observed between RSES and mortality (*p* = 0.862 after 1 month; p = 0.182 after 3 months; p = 0.886 after 6 months), after adjusting for age, sex, intervention, and MPI on admission.

### Secondary outcomes: rehospitalisation

The rehospitalisation rate following 1, 3, and 6 months was not found to be significantly associated with RSES (adjusted for age, intervention, sex, and MPI: p = 0.274, *p* = 0.754, and *p* = 0.972, respectively). Furthermore, the number of in-hospital days was not found to be significantly associated with RSES, after adjusting for age, sex, intervention, and MPI at admission. This was observed at one (*p* = 0.060), three (*p* = 0.366), and six months (*p* = 0.828, **Supp. Table 2**).

### Secondary outcomes: self-esteem and health status

Higher self-esteem (RSES) was nominally significantly associated with lower MPI at admission after adjusting for age, sex, and intervention (*p* = 0.002).

**Fig. 2:**
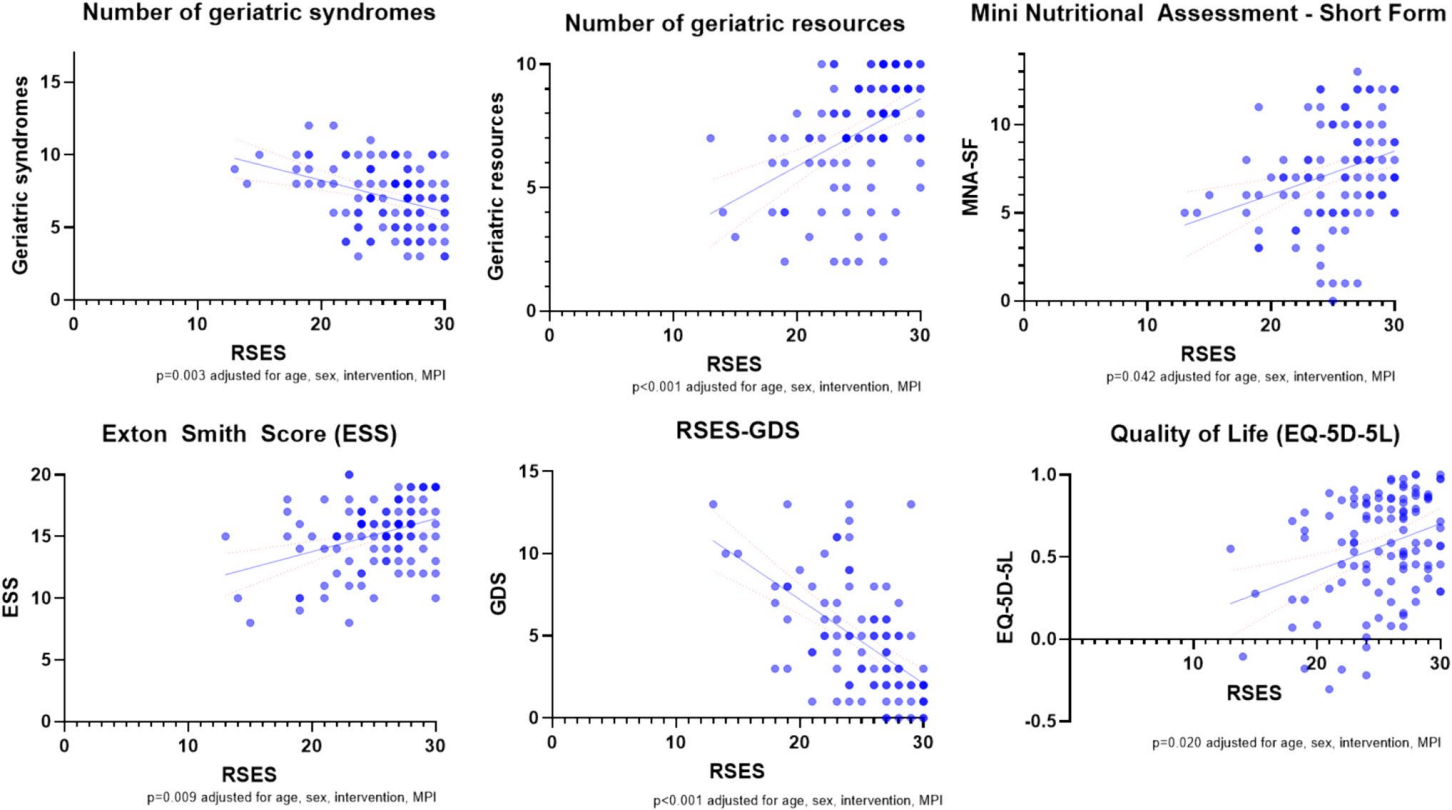
Association of RSES-based self-esteem with health status and PROMs. *Notes*: MPI = Multidimensional Prognostic Index, RSES = Rosenberg Self-Esteem scale, ESS = Exton Smith Scale; EQ-5D-5L = European Quality of Life 5 Dimensions 5 Level Version, GDS = Geriatric Depression Scale; MNA-SF: Mini Nutritional Assessment Short Form

Regarding MPI subdomains, RSES was nominally significantly associated with MNA-SF (*p* = 0.042, Fig. 2); patients with higher self-esteem had a better nutritional status, adjusted for age, sex, intervention, and MPI. Additionally, patients with higher self-esteem showed a nominally significantly lower pressure ulcer risk as indicated by ESS (*p* = 0.009, Fig. 2), after adjustment for age, sex, intervention, and MPI.

The number of geriatric resources and syndromes was nominally significantly associated with RSES (*p* < 0.001 and *p* = 0.003, adjusted for age, sex, intervention, and MPI, Fig. 2); patients with higher self-esteem exhibited a greater number of resources and a lower prevalence of syndromes, in comparison with patients with lower self-esteem.

The MPI at discharge was not found to be significantly associated with RSES (*p* = 0.232) when adjusted for age, sex, intervention, and MPI at admission.

### Secondary outcomes: self-esteem and PROMs

With regard to PROMs, RSES was nominally significantly associated with EQ-5D-5L (p = 0.020) and GDS (*p* < 0.001) on admission, adjusted for age, sex, intervention, and MPI—patients with higher self-esteem showed a nominally significantly higher quality of life and lower GDS score (Fig. 2).

The GDS at discharge (*p* = 0.010 adjusted for age, sex, intervention, MPI on admission, and GDS on admission) was nominally significantly associated with RSES (**Supp. Table 2**).

During the follow-up period, a nominal significant association was observed between GDS and RSES, adjusted for age, sex, intervention, MPI, and GDS on admission (1 month *p* = 0.908, 3 months *p* = 0.448, 6 months *p* = 0.970). 

With regard to the quality of life during the follow-up period, no significant association was observed between RSES and the aforementioned variables (*p* > 0.05 adjusted for age, sex, intervention, MPI, and EQ-5D-5L on admission).

## Discussion

The objective of this secondary analysis was to ascertain whether self-esteem is a predictor of mortality, geriatric conditions, readmission to hospital, and PROMs in older patients who have been hospitalised.

No association was observed between self-esteem and survival at six months post-discharge from hospital. In contrast, previous studies have demonstrated that lower self-esteem is associated with an elevated risk of mortality (Nirkko et al. 1982; Stamatakis 2004). The lack of confirmation of this result in the present analysis can be attributed to the relatively small number of subjects, with only 17% of patients in this study dying during the follow-up period. This conclusion is also supported by the fact that the MPI in the original publication of the RCT also failed to demonstrate a significant correlation with mortality after six months (Meyer et al. 2022). It may therefore be necessary for further longitudinal studies with a higher number of subjects and a longer follow-up period to clarify this question.

In regard to the findings of the secondary analysis, a notable correlation was observed between self-esteem and the geriatric profile, encompassing factors such as pressure ulcer risk (ESS), nutritional status (MNA-SF), the number of geriatric syndromes, and the utilisation of resources. This association remained nominal significant even when controlling for factors such as chronological age, biological sex, intervention, and MPI-biological age.

A notable correlation between self-esteem and frailty was initially demonstrated in the original publication of this RCT (Meyer et al. 2022). In this context, the secondary analysis of the RCT indicated that patients with higher self-esteem exhibit a nominally significantly reduced prevalence of geriatric syndromes. This result is consistent with previous publications, indicating that an increased number of geriatric syndromes is associated with lower life satisfaction in older adults (Yang et al. 2015). Nevertheless, this is the first study to demonstrate that patients with higher self-esteem have nominally significantly more geriatric resources. Although the prognostic significance of geriatric syndromes and resources has already been described in the literature (Meyer et al. 2020), this has not yet been demonstrated in relation to self-esteem. Previous studies have suggested that individual resources, such as financial resources, are nominally significantly associated with self-esteem (Borg et al. 2008). These findings are particularly intriguing when contemplating the potential of psychological interventions to bolster self-esteem, which could subsequently influence the geriatric profile of patients. In this context, a previous study has demonstrated that the maintenance of self-esteem and a sense of personal control can mitigate the effects of declining functioning in old age (Sargent-Cox et al. 2012). The initial publication of the RCT also demonstrated that a TIDP intervention resulted in improvements in frailty, self-esteem, and depressive mood at the time of discharge (Meyer et al. 2022). Further studies employing psychological interventions to bolster self-esteem and, consequently, resilience in older adults would be beneficial in elucidating the potential for enhancing the prognosis of older patients with multiple chronic conditions, thereby facilitating successful ageing (Hoogendijk et al. 2019; Dent et al. 2016, 2019).

Furthermore, it was demonstrated that the nutritional score MNA-SF and the pressure ulcer risk score ESS, in particular, are nominally significantly associated with self-esteem, independent of MPI-frailty. Previous research has consistently demonstrated that self-esteem is a reliable predictor of health-related behaviours (AbuSabha and Achterberg 1997), including those related to nutrition. One study identified self-esteem as a significant predictor of vitamin C and folate intake in women and folate intake in men. Additionally, the study found that higher self-esteem in men was associated with greater weekly consumption of vegetables and a more diverse range of vegetable choices (Schafer et al. 1999). Conversely, an intervention study utilising web-based nutrition education also demonstrated an improvement in self-esteem (Poddar et al. 2010). Other studies have demonstrated a correlation between elevated self-esteem and increased body weight (Megel et al. 1994). Although there is a paucity of studies examining this phenomenon in older adults, the results of these studies suggest that interventions designed to enhance self-esteem and promote an awareness of a balanced lifestyle may prove beneficial in improving the nutritional status and overall health of older individuals.

Furthermore, the secondary analysis indicated that patients at an elevated risk of developing pressure ulcers exhibited markedly diminished self-esteem. It is established that immobility and the plethora of physiological alterations associated with it can impact patients’ self-esteem, as self-esteem is constituted by an individual’s body image, performance, social functioning, and self-identification (Knight et al. 2019). For example, it has been documented that the body image of hospitalised patients undergoes changes during the course of their illness, which in turn leads to a decline in their self-esteem (Fingeret et al. 2014). The risk of pressure ulcer is increased by hospitalisation and bed rest, and these factors can also affect the performance self (inability to pursue interests and activities), the social self (interactions with friends and families as an important source of self-esteem), and the private self (dependence on others, loss of independence) (Knight et al. 2019). These factors are therefore closely intertwined with self-esteem and geriatric resources.

Although this secondary analysis demonstrated a nominal significant relationship between self-esteem and the subdomains ESS and MNA-SF of the MPI-biological age, this was not observed for other subdomains of the MPI, such as the SPSMQ as an indicator of cognition. This may suggest that self-esteem exerts a more pronounced impact on nutritional status and physical functioning/mobility than on other subdomains of biological age.

A further nominal significant finding of this secondary analysis is that PROMs including depression symptoms and quality of life are associated with self-esteem, independent of multidimensional frailty (MPI), age, gender, and intervention. 

The general finding that older adults with low self-esteem are at an increased risk of developing depression (Sowislo and Orth 2013; Doba et al. 2016) and experiencing a lower quality of life (Kermode and MacLean 2001) is not novel and has been well documented in various study populations. One theory that elucidates the relationship between low self-esteem and increased susceptibility to depression is the theory of *learned helplessness* (Brewin and Furnham 1986). This theory posits that negative experiences lead to the conviction that one has lost the ability to change one’s life situation and that one is responsible for this condition. As RSES was nominally significantly associated with GDS at admission and discharge, it is crucial to distinguish between low self-esteem and depression, as the latter is a neuropsychological diagnosis that requires neuropsychological assessment and treatment, including the possible use of medication if appropriate. In cases where self-esteem is low, it can be strengthened through occupational therapy, physiotherapy, or psychosomatic support. However, this is not a “disease” in the sense of depression, so intervention is desirable and also very useful in relation to PROMs, as this study shows, but this should not be seen as a disease treatment, but rather as a means of strengthening resources.

A general limitation of this study is that the assessment of self-esteem and others is inherently subjective, based on the statements of the patients surveyed. It is therefore evident that the results are contingent upon the context of the examination and the examiner’s influence. Nevertheless, these questionnaires on self-esteem and quality of life in advanced age remain the optimal survey instrument and are frequently employed in this context (Schofield 2018). While the sample size of 107 patients is sufficient for preliminary findings and to address associations, it may limit the generalisability of the results. A further limitation of this study is that self-esteem was only assessed using one scale, the RSES. In the future research, it would be beneficial to employ a range of instruments and conduct comparisons between them. Furthermore, given the number of variables included in the secondary analysis, it is possible that positive associations may be identified by chance. However, no significant correlations were found among the adjusting variables, indicating the absence of multicollinearity. Additionally, we acknowledge the limitations related to sample size and the number of variables. The model fit (Nagelkerke’s R^2^) showed low values not suitable enough for prediction, but the aim was not to build a predictive model. Rather, the focus was on exploring individual factors. Consequently, the interpretation of these results should be approached with caution. However, we already used the most restrictive statistical model possible, and the results are now named when appropriate “nominally significant”.

In general, the analysis revealed that self-esteem was not a predictor of mortality, rehospitalisation, or PROMs when considered independently of biological MPI age. This result may be explained by the relatively small number of subjects or the relatively short follow-up period. Conversely, an association between self-esteem and mortality, hospitalisation, quality of life, and depression symptoms has been demonstrated in other cohorts in the literature (Stamatakis 2004; Remm et al. 2023; Liu et al. 2020). Nevertheless, this has yet to be investigated specifically in older people or independently of underlying frailty, and should be the focus of further research with higher numbers of subjects.

A possible explanation for the result of this secondary analysis is that underlying frailty exerts the greatest predictive influence on health-related outcomes in older people.

 It may therefore be proposed that self-esteem should be regarded as a mediator rather than a predictor of health-related and geriatric outcomes. Nevertheless, self-esteem appears to be an important factor within the geriatric profile, encompassing all its associated resources and syndromes. Consequently, the incorporation of an additional standardised survey as part of a CGA is recommended. This is particularly pertinent to the MPI as a CGA, as it does not encompass any emotional or psychological subdomains of the older patient. Furthermore, there is a compelling need for additional intervention studies aimed at reinforcing self-esteem as a mediator, particularly in the context of patient-centred care.

## Conclusion

In conclusion, the current findings indicate that self-esteem may act as a mediator between health status, depressive symptoms, and quality of life in older patients undergoing acute hospital treatment, irrespective of frailty. In the light of the rapidly growing number of older patients receiving treatment outside geriatric settings and the pivotal role of self-esteem in PROMs, a systematic yet feasible approach to its assessment could prove beneficial in clinical practice. It is regrettable that many commonly used CGAs, such as the MPI, do not assess psychological domains such as self-esteem. As these results also indicate that self-esteem is a significant mediator of geriatric syndromes and resources, future studies could investigate whether self-esteem can enhance patient health outcomes through educational and rehabilitative programmes.

## Supplementary Information

Below is the link to the electronic supplementary material.

Supplementary file1 (DOCX 26 kb)

## Data Availability

The raw and/or processed data on which the secondary analysis conclusions are based, as well as the syntax required to reproduce the analyses, are available upon request from the authors.
