# Supplementary material for: Self-esteem is associated with health status and PROMs in advanced age independent of multidimensional frailty: secondary analysis from a RCT with 6-month follow-up
Source: Eur J Ageing. 2025 Oct 30;22(1):53. doi: 10.1007/s10433-025-00888-4 (PMC12572498; doi:10.1007/s10433-025-00888-4)
Supplement: Supplementary file 1 — Supplementary file1 (DOCX 26 kb) [file 10433_2025_888_MOESM1_ESM.docx]

**Supplementary**

**Supp. Table 1** **Association of geriatric morbidity with self-esteem**

| N=107 | **Median (IQR**) | **B** | **SD** | **p-value*** |
| --- | --- | --- | --- | --- |
| **MPI***,* n=105 | 0.50 (0.27) | -.013 | 0.004 | **0.002**** |
| **SPMSQ**, n=105 | 1 (2) | .021 | 0.051 | 0.685 |
| **MNA-SF***, mean (SD)* | 7.3 (2.9) | 0.144 | 0.070 | **0.042** |
| **ADL** | 4 (3) | -.034 | 0.033 | 0.311 |
| **IADL** | 4 (4) | -.042 | 0.046 | 0.371 |
| **ESS** | 15 (3) | .134 | 0.050 | **0.009** |
| **CIRS** | 6 (2) | .076 | 0.44 | 0.091 |
| **Geriatric resources** | 8 (3) | .218 | 0.053 | **<0.001** |
| **Geriatric syndromes** | 7 (4) | -,165 | 0.54 | **0.003** |
| **EQ-5D-5L** | 0.59 (0.50) | .018 | 0.008 | **0.020** |
| **VAS**, *n=103* | 50 (30) | 1.476 | 0.674 | **0.031** |
| **GDS**, *n=105* | 4 (4) | -.499 | 0.075 | **<0.001** |
| Table Note: MPI = Multidimensional Prognostic Index; SPMSQ = Short Portable Mental Status Questionnaire; MNA-SF = Mini Nutritional Assessment-Short form; ADL = Activities of Daily living; IADL = Instrumental Activities of Daily Living; ESS = Exton Smith Scale; CIRS = Cumulative Illness Rating Scale; EQ-5D-5L = European Quality of Life 5 Dimensions 5 Level Version; VAS = Visual Analogue Scale; GDS = Geriatric Depression Scale  *after linear regression analysis, results were adjusted for age, sex, intervention and MPI **after linear regression analysis, results were adjusted for age, sex and intervention | | | | |

**Supp. Table 2** **Association of follow-up results with self-esteem**

| N=107 | **Median (IQR)** | **B** | **SD** | **p-value^°^** |
| --- | --- | --- | --- | --- |
| **Discharge** | | | | |
| **MPI,** n=94 | 0.44 (0.14) | -.004 | 0.003 | 0.232 |
| **GDS***, n*=81 | 2 (3) | -.180 | 0.068 | 0.010 |

| **1-month Follow Up** | | | | |
| --- | --- | --- | --- | --- |
| **Alive***, n (%),* n=107 | 95 (89) | -.016 | 0.093 | 0.862 |
| **Rehospitalisation***, n (%), n=95* | 26 (27) | -.074 | 0.068 | 0.274 |
| **Rehospitalisation days**, n=94 | 0 (2) | -.643 | 0.337 | 0.060 |
| **GDS,** n=67 | 3 (3) | -.014 | 0.117 | 0.908 |
| **EQ-5D-5L,** n=75 | 0.65 (0.54) | -.146 | 0.164 | 0.377 |
| **VAS,** n=65 | 50 (28) | .325 | 1.169 | 0.782 |
| **3-months Follow Up** | | | | |
| **Alive**, *n (%),* n=105 | 87 (83) | -.114 | 0.086 | 0.182 |
| **Rehospitalisation***, n (%),* n=89 | 36 (40) | .024 | 0.076 | 0.754 |
| **Rehospitalisation days**, n=84 | 0 (3) | .415 | 0.457 | 0.366 |
| **GDS,** n=60 | 3 (3) | -.100 | 0.131 | 0.448 |
| **EQ-5D-5L,** n=61 | 0.74 (0.48) | -.001 | 0.014 | 0.951 |
| **VAS,** n=61 | 60 (25) | .648 | 0.962 | 0.504 |
| **6-months Follow Up** | | | | |
| **Alive***, n (%),* n=103 | 85 (83) | -.121 | 0.086 | 0.886 |
| **Rehospitalisation**, *n (%),* n=88 | 45 (51) | -.029 | 0.083 | 0.972 |
| **Rehospitalisation days**, n=83 | 0 (0) | .109 | 0.498 | 0.828 |
| **GDS,** n=61 | 4 (3) | -.005 | 0.140 | 0.970 |
| **EQ-5D-5L,** n=71 | 0.61 (0.49) | -.027 | 0.014 | 0.061 |
| **VAS,** n=59 | 60 (30) | 2.531 | 1.095 | 0.025 |
| Table note: MPI = Multidimensional Prognostic Index; GDS = Geriatric Depression Scale; EQ-5D-5L = European Quality of Life 5 Dimensions 5 Level Version; VAS = Visual Analogue Scale. *after linear/logistic regression analysis, results were adjusted for age, sex, intervention and MPI on admission and if assessed the responding score on admission | | | | |
